# H—to Annie

**Published:** 1891-12

**Authors:** 


### H—— TO ANNIE.

(On the Nineteenth Anniversary of their Wedding).

Ay, let the day be ours, Annie,
And ours the memories dear,
That reach away
From our marriage day,
To this, the nineteenth year.

We've turned another leaf, Annie,
Of life's eventful tome,
The "Pages" twain
May love retain,
With blessings on our home.

'Neath sunny skies and sad, Annie,
We've kept our onward way,
Unshaken still
The single will,
That joins our hands to-day.

Be all our days to come, Annie,
As peaceful as the past,
And Heaven forefend
That they should end
Without some good, to last.

We've learned that Heaven is near, Annie,
And open wide the door,
That friends we knew,
Though passed from view,
Are with us as before :

That death is only change, Annie,
From this to higher states,
For the lowliest one
Beneath the sun,
Some loving angel waits.

NEW YORK, Nov. 9, 1891.

